# Chain length of bioinspired polyamines affects size and condensation of monodisperse silica particles

**DOI:** 10.1038/s42004-021-00595-y

**Published:** 2021-11-19

**Authors:** Sai Prakash Maddala, Wei-Chih Liao, Rick R. M. Joosten, Mohammad Soleimani, Remco Tuinier, Heiner Friedrich, Rolf A. T. M. van Benthem

**Affiliations:** 1 Laboratory of Physical Chemistry, Department of Chemical Engineering and Chemistry & Institute for Complex Molecular Systems, Eindhoven University of Technology, P.O. Box 513, 5600 MB Eindhoven, The Netherlands; 2 Center for Multiscale Electron Microscopy, Eindhoven University of Technology, Groene Loper 5, 5612 AE Eindhoven, The Netherlands; 3 DSM Materials Science Center, 6167 RD Geleen, The Netherlands

**Keywords:** Nanoparticles, Biophysical chemistry, Cryoelectron microscopy, Bioinspired materials, Organic-inorganic nanostructures

## Abstract

Polyamines play a major role in biosilicification reactions in diatoms and sponges. While the effects of polyamines on silicic acid oligomerization and precipitation are well known, the impact of polyamines chain length on silica particle growth is unclear. We studied the effects of polyamine chain length on silica particle growth and condensation in a known, simple, and salt-free biphasic reaction system; with tetraethyl orthosilicate as organic phase and polyamine dissolved in the aqueous phase. The particles at various growth stages were characterized by Cryo- Transmission Electron Microscopy, Scanning Electron Microscopy, Thermogravimetric Analysis, Zeta Potential, and solid-state NMR analysis. Polyamines were found co-localized within silica particles and the particle diameter increased with an increase in polyamine chain length, whereas silica condensation showed the opposite trend. Particle growth is proposed to progress via a coacervate intermediate while the final particles have a core shell structure with an amine-rich core and silica-rich shell. The results presented in this paper would of interest for researchers working in the field of bioinspired materials.

## Abstract (web summary)

Polyamines play an important role in biogenic silica formation, however, their effects on silica particle size and condensation have not been well described. Here the authors show that bioinspired polyamine length affects silica particle size and extent of condensation.

## Introduction

Silica is the most abundant mineral in the Earth’s crust. Silica, in its amorphous form, is found in a variety of organisms ranging from algae (diatoms) to plants and sponges, where it has functions that range from structural material, light harvesting, stress tolerance and others^1–3^. Biogenic silica displays morphological complexity^4^ unmatched by current synthetic silica production techniques, it is deposited in the form of 30 to 100 nm particles^5,6^. Silica particles in nature are produced at ambient conditions, in an aqueous environment, aided by long chain polyamines (LCPA)^7,8^. This presents a substantial advantage over conventional Stöber method^9^, where large amount of organic solvents such as ethanol are used. Unlike conventional silica particles produced from Stöber process^10^, biogenic silica particles are silica-biomolecule composites^7,11^. Development of novel bioinspired particle size control strategies could further inspire the production of new functional materials.

LCPA and their synthetic analogues such as ethylenamines^12,13^, propylenamines^14,15^, polyallylamine^16^, polylysine^17^ have been shown to influence silicic acid condensation positively and negatively. Orthosilicic acid condenses to form silica particles with broad size distribution^13,14,18,19^ at slightly acidic pH (pH between 5.5 and 6.8) in presence of polyamines. While the above studies demonstrate a clear correlation between silica yield and the presence of polyamines, they did not demonstrate any correlation between silica particle size and polyamine structure. The silica particle formation in these studies was probably affected by two factors viz., pH and secondary ions. At pH close to neutral, silicic acid undergoes rapid condensation^20^, which likely resulted in rapid precipitation of the particles, resulting in a poor control of particle size. The particle syntheses were typically carried out in sodium phosphate buffers. Sodium ions^21^ have been shown to affect silicic acid condensation. Phosphate ions phase separate polyamines^19,22^, resulting in rapid precipitation of silica particles. Silica formation^23^ of under biomimetic conditions has been described using two contradictory mechanisms; the first one involves phosphate ion assisted phase separation (due to coacervation of polyamines with phosphate), which then act as templates for silica production^5,18,19,24,25^. Here silicic acid oligomers interact with polyamines primarily by ionic interactions. The second mechanism involves phase separation of silicic acid oligomers and polyamines, and according to this mechanism silicic acid molecules interact with polyamines by a combination of ionic, hydrogen bond and hydrophobic interactions^13–15,26^. In this mechanism secondary ions such as phosphate act buffers and do not significantly influence silica formation. However, it has recently been discovered^27^ that phosphate ions bind strongly to polyamines, which would prevent silicic acid molecules from directly interacting with polyamines. It is therefore unclear whether the observed effects on silica particle morphology are the direct result of polyamine–silicic acid interactions or the effect of polyamine–phosphate–silicic acid interactions.

**Fig. 1:**
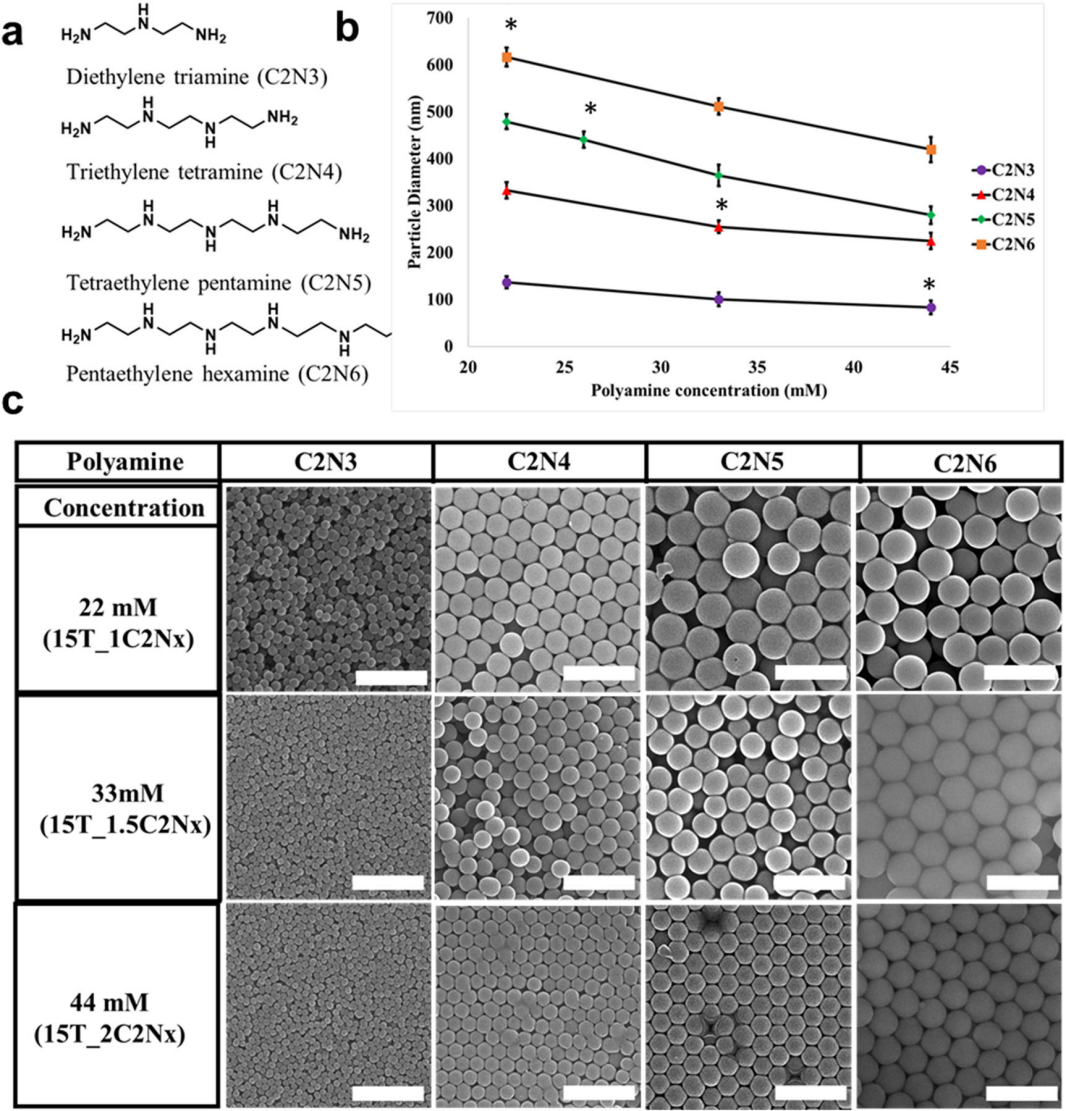
Bioinspired silica synthesis using polyamines of different chain lengths and concentrations. **a** Molecular structures of polyamines. **b** Plot of average silica particle diameter (*n* = 100; error bars represent stand deviation) obtained using SEM images of particles grown in presence of different polyamines and at different polyamine concentrations (data points marked with * correspond to the particle diameters obtained at a constant Si:N ratio of 2.5:1), and **c** SEM images of final particles grown at various polyamine concentrations (22–44 mM (the corresponding mole ratios in brackets)) using 330 mM silicic acid concentration in aqueous phase. Scale bars 1 µm.

**Fig. 2:**
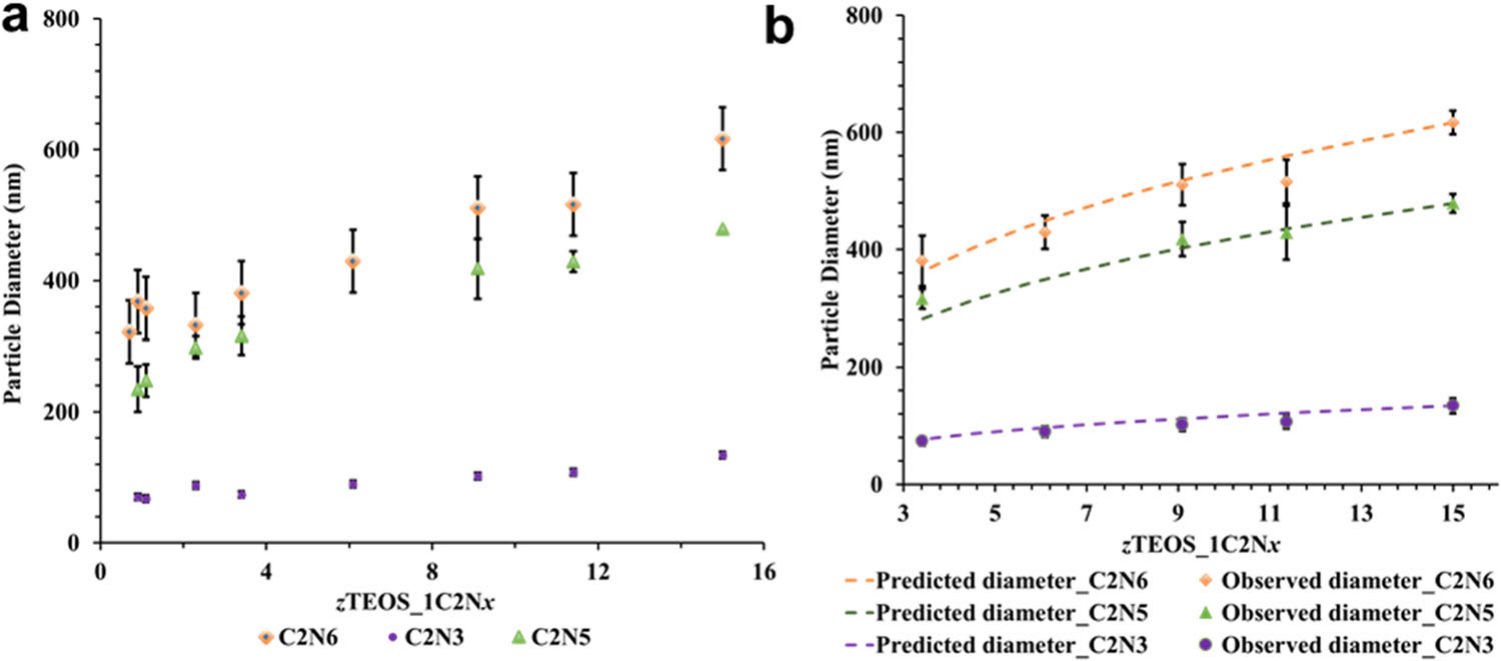
Bioinspired silica synthesis using polyamines of different chain lengths and at varying *z*TEOS_1C_2_N_x_ mole ratios. **a** Average diameter of silica particles produced in presence of constant polyamine concentration of 22 mM at 60 °C (error bars represent stand deviation); **b** Observed and predicted (dashed line) average diameters of silica particles grown at higher *z*TEOS_1C_2_N_x_ mole ratios, *z* = 3.4–15). Average diameters were calculated from SEM images by measuring 100 particles using an in-house MATLAB script (error bars represent standard deviation).

**Fig. 3:**
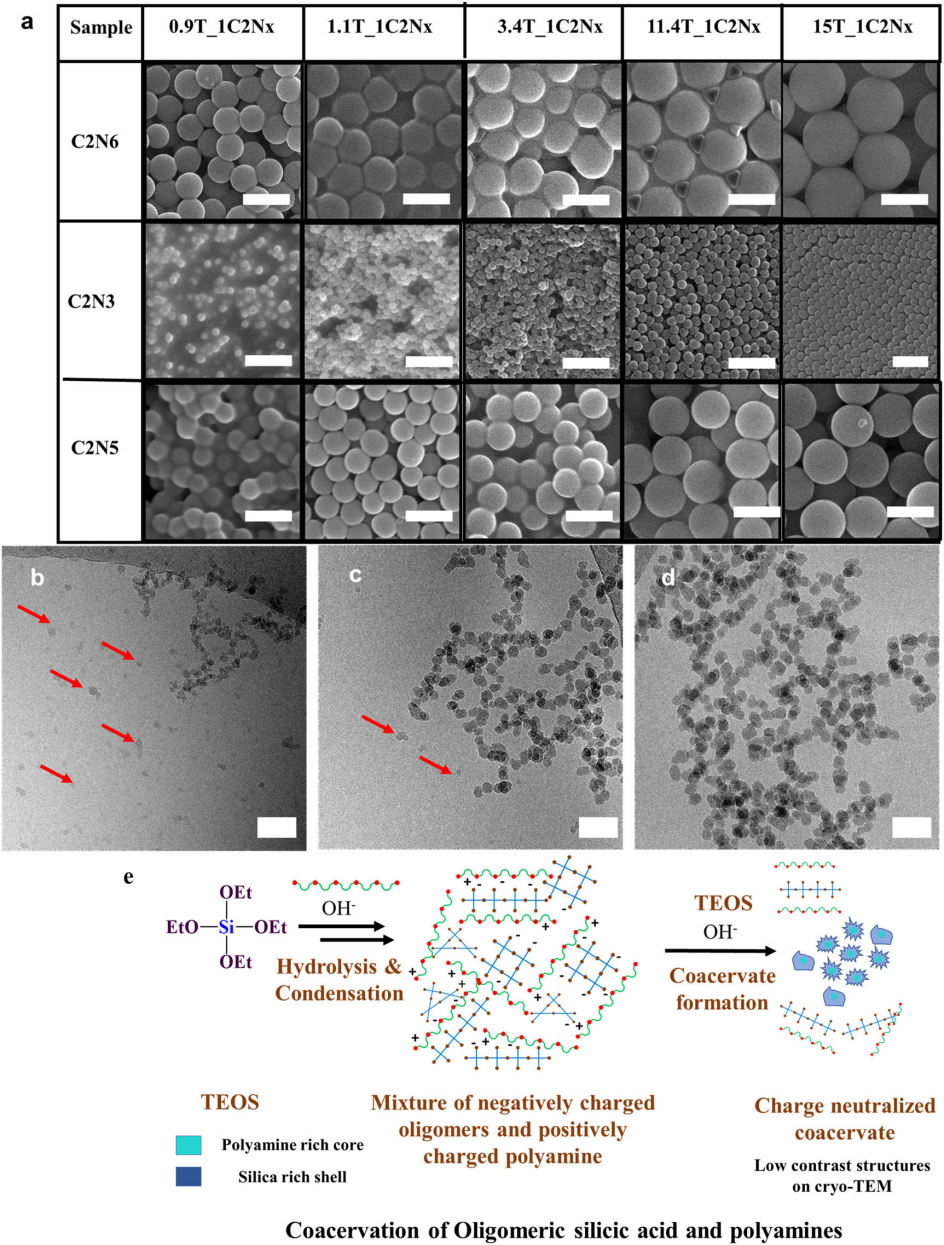
Electron Microscope images of bioinspired silica synthesis using polyamines of different chain lengths and at varying *z*TEOS_1C_2_N_x_ mole ratios. **a** SEM images of Silica particles grown in presence of 22 mM polyamines at various amounts of TEOS (scale bar 500 nm); Silica particles produced in presence of 22 mM C_2_N_3_ at 0.70TEOS_1C_2_N_3_ mole ratio samples vitrified at: **b** 60 min, **c** 75 min, **d** 90 min (scale bar 50 nm). Low contrast structures are highlighted with red arrows. **e** Schematic representation of coacervate formation between oligomeric silicic acid and polyamines by charge matching.

**Fig. 4:**
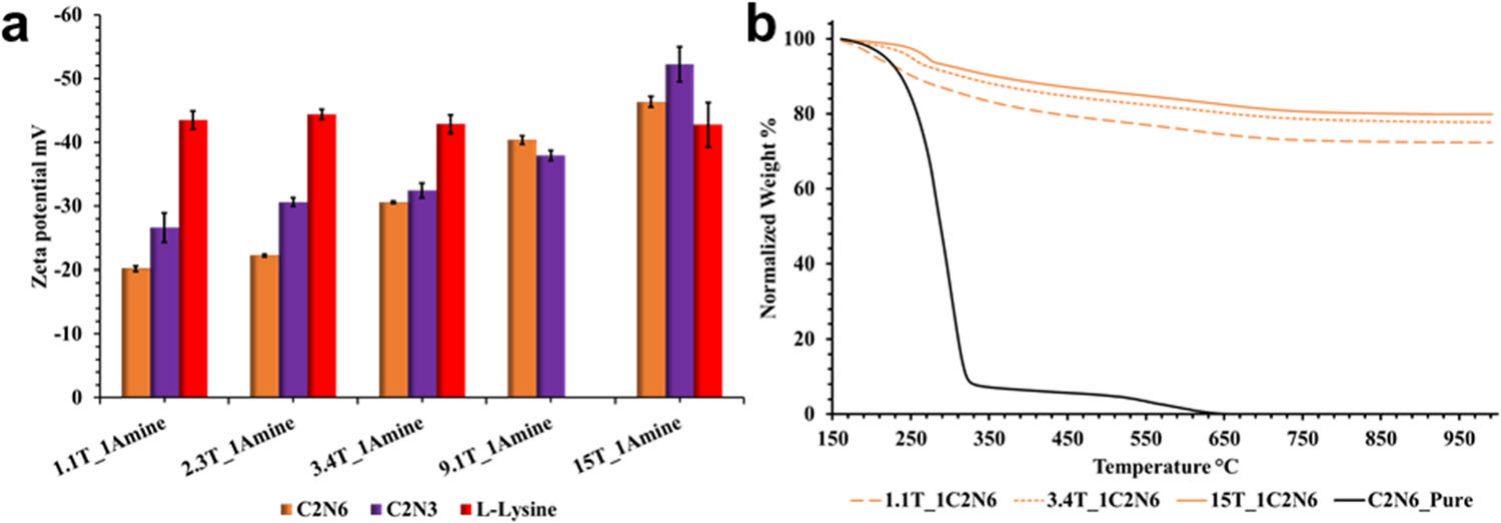
Zeta potential and TGA of polyamine–silica particles. **a** Average zeta potential obtained for silica particles (1.5 mg/mL) grown at different mole ratios. Samples were measured thrice (*n* = 3) in 22 mM polyamine solution (error bars represent standard deviation). **b** Normalized TGA curves of silica particles grown at increasing *z*TEOS_1C_2_N_6_ mole ratio obtained by heating the samples in air. Samples were heated from RT to 1000 °C at 20 °C/min. Sample weight-loss was normalized from 160 °C to account for loss of adsorbed water.

**Fig. 5:**
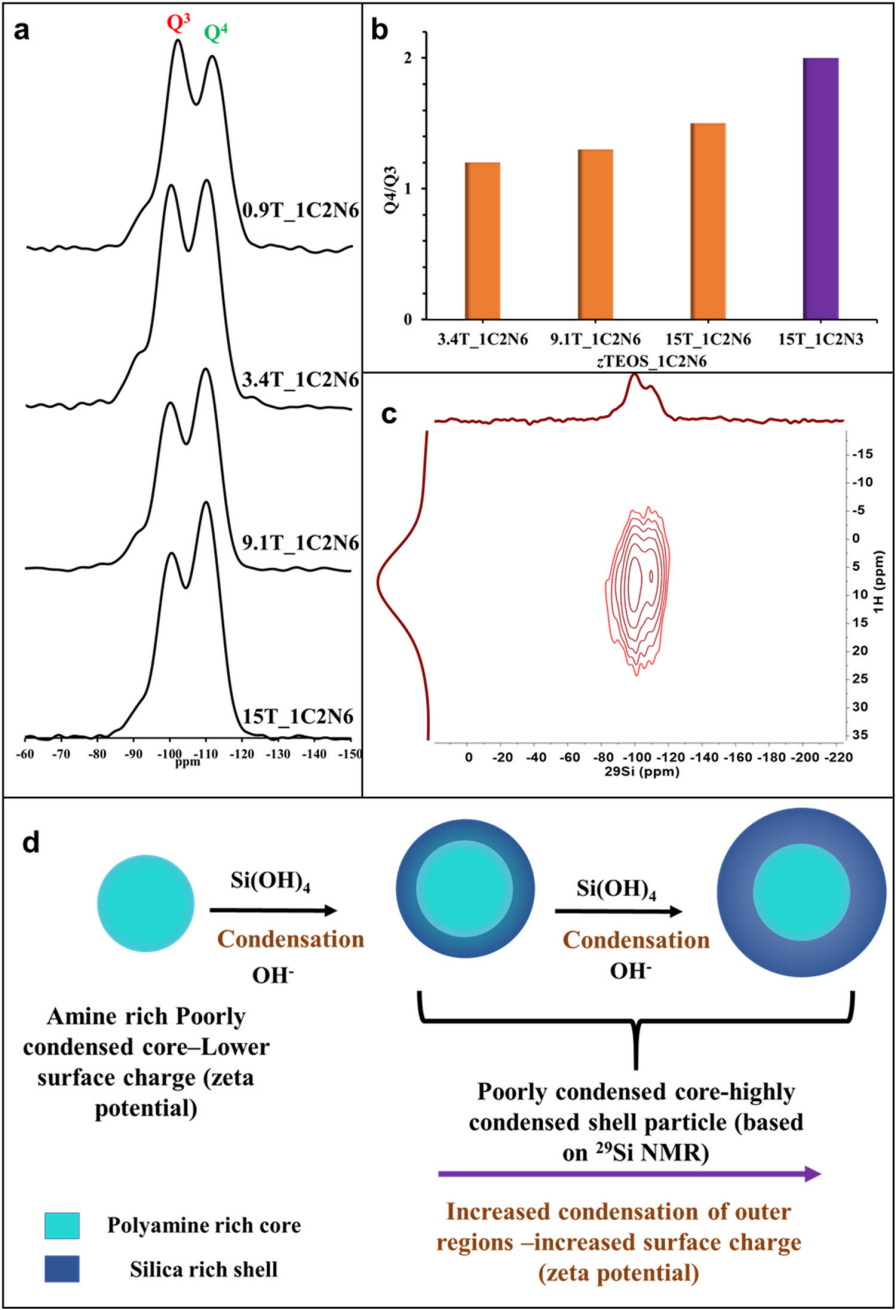
^29^Si Solid State NMR silica particles produced at various *z*TEOS_1C_2_N_6_ mole ratios. **a** ^29^Si Solid state NMR spectra of silica particles produced in presence of 22 mM C_2_N_6_ obtained at various mole ratios. **b** *Q*^4^/*Q*^3^ ratios of silica particles obtained from deconvolution of ^29^Si SSNMR spectra. **c** 2D ^29^Si–^1^H HETCOR NMR with CP contact time of 3 ms of silica particles produced at 15T_1C2N6 mole ratio. **d** Schematic representation of particles at different stages of growth.

In order to probe the specific effects of polyamine–silicic acid interactions on silica particle formation in the absence of secondary ions, we investigated the influence of adding different polyethyleneamines^14^, on the resulting silica particle diameter using the so-called Yokoi method^28,29^. We chose this system because it is simple, has been studied widely, and affords highly monodisperse spherical silica nanoparticles. The particle formation mechanism under these conditions has been well established^29–32^. The Yokoi method involves silica particle growth in a biphasic system, consisting of an aqueous phase at basic pH (pH > 9) and tetraethyl orthosilicate (TEOS) as organic phase. Basic pH aides in steady hydrolysis of TEOS^28^, releasing silicic acid into the aqueous phase (see the “Methods” section and Supplementary Methods). At this pH silicic acid undergoes slow condensation^20,33^, which provides greater control over particle size. As a result, the particle synthesis takes a much longer duration (several hours) compared to those produced from silica precursors like hydrolyzed TMOS and hypervalent silicon complexes^14,18^. The polyamines used in this study (see their molecular structures in Fig. 1a) were chosen due to their similarity to the polyamines found in diatoms^7^ and their ability to catalyse the oligomerization of silicic acid^14^. More specifically, the effect of polyamine length on silica particle formation and growth was investigated. Previous studies^13^ showed a qualitative relationship between silica particle size and polyamine length, but due to broad particle size distribution and the formation of fused particles it is difficult to determine the effect of polyamines on silica growth and condensation. Unlike previous investigations, the “salt-free” aqueous phase here only contained dissolved polyamines, the reaction was therefore studied without the interference of secondary ions (like sodium and phosphate ions) or phosphate-ion-induced phase separation of amines^25^. The reaction conditions used in this study yielded highly monodisperse silica particles in all cases (Fig. 1b, c), which allowed us to compare the effects of increasing polyamine chain length on silica particle size. We demonstrate that the silica particle size increases with increasing chain length accompanied by a decrease in the degree of condensation (Fig. 5). We also investigated the effects of varying polyamine and TEOS stoichiometries (Figs. 2–5) on particle growth, polyamine incorporation, zeta potential and degree of condensation. We determined that the particles have a “core–shell” structure with a polyamine-rich core and silica-rich shell. We also proposed a mechanism to explain particle formation in presence of polyamines.

## Results

Silica particle synthesis was carried out at 60 °C using a biphasic reaction system with TEOS (organic phase) as silica precursor and a polyamine solution at pH 10.9 as the aqueous phase (experiment details are provided in the “Methods” section and Supplementary Methods). The reaction was carried out for 16 h. The total silicic acid concentration in the aqueous phase (particles + soluble silicic acid) was 330 mM. Samples are labelled with the following^14^ code: *z*T_*y*C_2_N_*x*_, where *z* is number of moles of TEOS added to the reaction, *y* is the number of moles of polyamine, C_2_ stands for the ethylene linkers and *x* for the number of nitrogen atoms in a polyamine molecule. *z*T_*y*C_2_N_*x*_ refers to the mole ratio of TEOS to polyamine.

### Effect of polyamines on silica particle size

Silica particles synthesized in presence of simple organic (ethylamine, ethylene diamine, triethylamine, l-lysine) and inorganic (sodium hydroxide and ammonia) bases at pH 11 gave 10–20 nm particles (Supplementary Note 2). However, when the particles were grown in presence of polyamines two trends were observed. First the particle diameter increased with increasing polyamine chain length, with particle diameter, measured from SEM images, (Fig. 1b, c and Supplementary Note 3)) ranging from 137 ± 13 to 617 ± 20 nm for C_2_N_3_ and C_2_N_6_, respectively, at 22 mM polyamine concentration. Second, silica particle diameter was found to vary inversely with the polyamine concentration, e.g. an increase in C_2_N_6_ concentration from 22 to 44 mM resulted in a particle diameter decrease from 617 ± 20 to 424 ± 28 nm. Similar trends were observed for all other polyamines (Fig. 1b, c and Supplementary Note 3). Notably, the same trends were observed even when Si:N ratio was kept constant at 2.5:1 (Fig. 1b) indicating a direct relationship between polyamine length and silica particle diameter.

### Time-resolved growth of silica particles

Silica particle formation, under the present reaction conditions broadly, involves two stages, viz, emulsification (due to stirring) and hydrolysis of TEOS (organic phase) to silicic acid oligomers into the aqueous phase; where subsequent condensation, nucleation, and growth of silica particles^29^ take place. The reaction stirring rate (900 rpm) was optimised to ensure reaction completion and to obtain a monodisperse particles. When particle synthesis is catalyzed by simple amine bases such as l-lysine, the amines act as counterions, are restricted to the surface of the silica particles^29,34^, and thus are not included within the silica matrix. In the presence of polyamines, however, we must consider co-precipitation of the polyamines and the polysilicic acid oligomers as well, since polyamines are found to be intimately mixed with silica in diatoms^7,35^.

Silica particles were grown at increasing TEOS to polyamine mole ratios. The duration of the individual reactions was optimised to ensure that the silica particle formation was complete. This experimental setup allowed us to isolate and characterize particles produced at different TEOS to polyamine (*z*T_*1*C_2_N_*x*_) mole ratios. Between 0.90T_1C_2_N_*x*_ and 2.3T_1C_2_N_*x*_ mole ratios, using C_2_N_5_ and C_2_N_6_, the particles had somewhat broader size distributions as compared to the particles grown in presence of C_2_N_3_ (Figs. 2 and 3a) as evidenced from the particle size standard deviations (Supplementary Table S4a). In the presence of C_2_N_6_, at 0.90T_1C_2_N_6_ mole ratio 368 ± 56 nm diameter particles were observed, and a further increase in TEOS amount gave fluctuating particle diameters of 358 ± 41 and 333 ± 49 nm at 1.1T_1C_2_N_6_ and 2.3T_1C_2_N_6_, respectively. When C_2_N_5_ was used as base, particle diameters of 235 ± 35, 248 ± 25, 298 ± 76 nm were observed at 0.9T_1C_2_N_5_, 1.1T_1C_2_N_5_ and 2.3T_1C_2_N_5_, respectively (Figs. 2,  3a and Supplementary Table S4a). The relatively constant particle size despite an increase in the absolute concentration of silicic acid indicated that the particle numbers increased with TEOS concentration.

From 3.4T_1C_2_N_*x*_ onwards, particles produced in presence of C_2_N_5_ and C_2_N_6_, the particle diameters no longer fluctuate; instead, they steadily increase with increasing *z*TEOS_C_2_N_*x*_ mole ratio while a decrease in the particle size standard deviation was observed, i.e., a decrease in particle size polydispersity (Figs. 2,  3a and Supplementary Table S4a). During aqueous silica particle synthesis, the particle numbers remain constant after an initial burst^29,30^. This allows for the calculation of particle diameters at mole ratios between 3.4T_1C_2_N_*x*_ and 15T_1C_2_N_*x*_ (Supplementary Note 4; Supplementary Eq. (1)). Figure 2b shows that the predicted particle diameters are in close agreement with the observed particle diameters for all the polyamines. This implies that the particles have a nucleation phase up to 3.4T_1C_2_N_*x*_ where the particle “core” is produced, and further TEOS addition leads to particle growth without any additional nuclei formation. Particles grown in presence of C_2_N_3_ at 0.90T_1C_2_N_3_ and 1.1T_1C_2_N_3_ mole ratios gave 70 ± 7, 68 ± 6 nm particle diameters, respectively. The particle diameters steadily increased thereafter. Interestingly, there was no change in the size dispersity of the particles grown in presence of C_2_N_3_.

Silicic acid has a solubility limit of 15 mM (at pH ≥ 10 and 60 °C)^30,33^. When simple amine bases are used, the first particles are observed at this concentration^30^. In presence of C_2_N_6_ irregularly shaped 100–200 nm structures were observed at 0.50T_1C_2_N_6_ mole ratio (Supplementary Fig. S5a), which corresponds to 10 mM silicic acid concentration. A small increase in mole ratio to 0.57T_1C_2_N_6_ (12.5 mM silicic acid concentration) gave well-defined 200–400 nm particles (Supplementary Fig. S5c). However, particles were not observed at the same mole ratios when C_2_N_3_ was used. The results presented here demonstrate either earlier oligomerization^36^ (Fig. 3e) (see “Discussion” section for further details)-like precipitation in presence of C_2_N_6_ compared to C_2_N_3_ and simple bases. In presence of C_2_N_3_ the first particles were observed at 0.70T_1C_2_N_3_ mole ratio (15 mM silicic acid concentration, Supplementary Fig. S5f). Due to their small size (35 ± 5 nm), they were well suited for time-resolved cryo-TEM investigation. Samples were vitrified at 60-, 75- and 90-min time points. At 60 min (Fig. 3b and Supplementary Fig. S6) high contrast solid particles (22 ± 5 nm) with a jagged surface and irregularly shaped low contrast structures were observed. At 75 min (Fig. 3c) the high contrast particles showed a small increase in particle size (31 ± 6 nm) and appeared smoother than the particles at 60-min time point (statistical significance measured using two-tailed *t*-test; *p* < 0.001*; df* = 198). There were noticeably fewer low contrast structures. At 90 min (Fig. 3d) the shape of high contrast particles became much more defined (35 ± 5 nm) and monodisperse (due to reduction in percentage standard deviation from 23% at 60 min to 14% at 90 min), and low contrast structures were completely absent.

### Characterization of silica particles

The presence and co-localization of polyamines in the particles was confirmed qualitatively by SEM–energy dispersive X-ray spectroscopy (EDS) (Supplementary Note 7) and IR spectroscopy (Supplementary Note 8). EDS elemental maps collected for whole particles (Supplementary Fig. S7a) and FIB milled (Supplementary Fig. S7b) silica particles produced in the presence of 22 mM C_2_N_6_ clearly showed not just the presence of, but true internal co-localization of polyamines and silica. The FIB-SEM image clearly shows that the particles are not hollow. IR spectra were collected for freeze-dried silica particles produced in presence of 22 mM C_2_N_6_. Si–O–Si symmetric and anti-symmetric stretching vibrations at 800 and 1044 cm^−1^, respectively, and Si–OH bending vibrations^37^ at 952 cm^−1^ were observed (IR spectrum of silica produced in the absence of polyamines provided for reference). Broad peaks at 1366 and 1456 cm^−1^ which correspond the C–N stretching vibrations^38^ and peak at 2969 cm^−1^ correspond to C–H stretching vibrations were observed as well. The presence of peaks corresponding to the polyamines clearly indicated their presence along with the silica in the particles. (Supplementary Note 8).

Zeta potential measurements were carried out on particle dispersions prepared in the presence C_2_N_6_ and C_2_N_3_ using different amounts of TEOS and in presence of 22 mM polyamine (Fig. 4a). All the particles had a negative charge, as expected for silica particles at high pH^39^. Increasing the amount of TEOS resulted in an increase the magnitude of the zeta potential of the particles in both cases. In contrast, particles produced in presence of l-lysine did not show such an increase (Fig. 4a), the particles had a zeta potential of around −42 mV at all concentrations.

Thermogravimetric analysis (TGA) was performed on freeze-dried silica powders by heating in air to 1000 °C. The weight loss curves were normalized to account for the loss of adsorbed water^40^. The weight loss observed between 160 and 650 °C corresponded to loss of incorporated organic molecules (C_2_N_6_) (Fig. 4b)^41,42^ demonstrating that increasing the amount of TEOS results in higher amount of inorganic content, i.e., greater silicification. The TGA curves of particles produced at 15T_1C_2_N_*x*_ mole ratio (Supplementary Fig. S9), showed that in presence of longer polyamines a higher weight percentage of organic molecule was incorporated compared to those produced in presence of shorter polyamines, as is expected based on the molar mass of the polyamines. Based on the TGA data, the Si:polyamine ratios of around 1:0.06 were obtained in all cases, confirming that around 90 percent of the molar amount of polyamines added to the reaction mixture were incorporated into the particles.

Solid state magic angle spinning (MAS) NMR measurements were carried out on freeze-dried particles to reduce inter and intraparticle silanol condensations during sample preparation.^1^H NMR analysis on 617 ± 20 nm particles (15T_1C2N6) produced in presence of C_2_N_6_ gave a broad spectrum (Supplementary Fig. S10a). The peak centred at 6.6 ppm was assigned to protonated primary amine^43^ (R-NH_3_^+^) and the peaks centred at 3.6 and 1.2 ppm were assigned to physisorbed water and silanols^44^. ^29^Si quantitative-MAS NMR was collected on particles at four mole ratios (Fig. 5a) prepared in the presence of C_2_N_6_. *Q*^4^, *Q*^3^ and *Q*^2^ peaks (Supplementary Fig. 10b) at −110, −100 and −91 ppm, respectively^45^ (Supplementary Table S10) were observed. The peak positions and relative areas were obtained by deconvolution of the spectra^46^ based on Gaussian distribution function. The deconvoluted spectra showed that with increasing particle diameter, the relative amount of, fully condensed silica or *Q*^4^ species increased and the amounts of, partially condensed, *Q*^2^ and *Q*^3^ species decreased. Particles produced at 0.9T_1C_2_N_6_ mole ratio had around 47% *Q*^4^ species. This value increased to 56% *Q*^4^ for the 617 ± 20 nm (15T_1C_2_N_6_). The decrease in *Q*^3^ and *Q*^2^ species indicated a reduction in silanol density with increasing particle diameter. Consequently, the *Q*^4^/*Q*^3^ ratio increased from 1.1 to 1.5 (Fig. 5b and Supplementary Table 10) with increasing particle diameter. The particles are not hollow (Supplementary Fig. S7b). The particles therefore have a “core–shell” structure composed of a “core” with lower condensation and high organic content and a “shell” with higher condensation (Fig. 5d). ^29^Si NMR spectrum obtained from particles grown at 15T_1C_2_N_3_ mole ratio (137 ± 13 nm in diameter) (Supplementary Fig. S10c and Supplementary Table S10) showed a substantial increase in the relative amount of *Q*^4^ species (63%) resulting in *Q*^4^/*Q*^3^ ratio of 2.0 (Fig. 5b and Supplementary Table 10). ^29^Si−^1^H heteronuclear correlation (HETCOR) measurement (Fig. 5c and Supplementary Fig. S11) on particles obtained from C_2_N_6_ at 15T_1C_2_N_6_ mole ratio showed a stronger correlation between ^29^Si-*Q*^3^ silanols (−100 ppm) and ^1^H-ammonium peak at 6.6 ppm and acidic protons with higher chemical shift values centred at ca. 8 ppm, and a weaker correlation between the ^1^H ammonium peak and ^29^Si *Q*^4^ species (−110 ppm) is observed as well.

## Discussion

These experimental observations can be explained by considering interactions between silicic acid oligomers and polyamine, in terms of p*K*_a_, number of binding sites and bonding types. Silicic acid oligomers have p*K*_a_ values between 8 and 10 and are therefore negatively charged at the reaction pH^26,47^. The polyamine molecules are positively charged. However, the amine groups in the polyamine molecules are known to resist protonation when their neighbouring amines are protonated^48^. This resistance to protonation is further reflected in the different p*K*_a_ values of the consecutive (protonated) amine groups in polyamines (Supplementary Note 12)^49^. Increasing polyamine length results in an increase in the number of unprotonated amines^14,18^ at the pH of our experimental conditions. The presence of the unprotonated amines has been demonstrated to increase the rate of oligomerization of silicic acid^14^. By binding to the silanols, the unprotonated amines can achieve localized activation of silanols by deprotonation and aid in their nucleophilic attack on silicic acid molecules^15^ resulting in faster oligomerization. Due to the presence of a large number of unprotonated amines, longer polyamines provide a water-free microenvironment, which is more efficient at driving for proton/donor acceptor mechanism^14^. Longer polyamines are thus able to accelerate oligomerization reactions^36^. The positively charged polyamines bind to the negatively charged silicic acid oligomers not only via their multiple amine groups but also in multiple bonding modes: via ionic, van der Waals and hydrogen bond interactions. Even at high pH, amines bind strongly to silica due to increased silanol dissociation^50^. Computational studies demonstrated that the ionic interactions are dominant and result in an irreversible attachment of polyamines to negatively charged silicic acid oligomers^51^. In the context of biomimetic silica formation, the term coacervation of has been used to either describe polyamine-phosphate templates or to describe silicic acid–polyamine complexes. In this paper, we use the term coacervation to describe the latter.

The interactions between multiple negatively charged silicic acid oligomers and multiple positively charged polyamines result in charge-neutralized coacervate-like structures. Longer polyamines can effectively bridge silicic acid oligomers leading to formation of larger coacervates^51^. The strong affinity^52,53^ between silanols and amines coupled with the multiple binding sites of the polyamines prevents dissociation and ensures the stability of the coacervates^36^.

The results in this paper show that the longer polyamines initiate nucleation at an earlier stage compared to shorter polyamines and bind more strongly in the coacervate with silicic acid oligomers due to an increased number of binding sites; while this offers a qualitative explanation for the observed increase in particle diameter (Figs. 1b, c, 2 and 3a and Supplementary Note 4), for a more comprehensive understanding we also need to consider their effects on the condensation state of the nascent particles. Due to the effective charge neutralization in these coacervates, they readily aggregate to larger, but still undefined structures, until they reach a critical size. The coacervate-aggregates gradually develop a negatively charged surface due to ongoing silanol condensations which result in the formation of more acidic silanols at the expense of less acidic ones^26^. An electrical double layer is formed following the build-up of sufficient net negative surface charge and the mobility of the positive counter ions. The size and magnitude of this double layer increases the colloidal stability of the particles. Once the repulsive maximum of the total pair interaction exceeds a few kT particle growth is arrested^54^. Further intracoacervate silanol condensation results in the formation of particles. Coacervate to particle transition has been proposed as a key step in biosilica formation as well^16^.

The nature of the polyamine further affects the intraparticle condensation. As evidenced from the ^29^Si MAS NMR results (Supplementary Table S10), longer polyamines act as effective steric barriers and therefore slowdown intraparticle silanol condensation more effectively than shorter polyamines. Thus, longer polyamines lead to more poorly charged aggregates and nucleate at larger critical particle size (Supplementary Figs. S5a, S5c). The charge distribution also affects the particle growth rate. Smaller particles, have lower absolute charge (Fig. 4a), due to their high amine content (Fig. 4b) and as a result these particles grow at a faster rate due to aggregation with coacervates leading to monodisperse particle size distributions^55^. This can be clearly seen in Fig. 3b–d where low density coacervates are observed at 60-min time point along with polydisperse particles and at 90-min time point only well-defined monodisperse particles are observed. Following the gradual depletion of the polyamines from solution, coacervate to particle nucleation is gradually taken over by particle growth via the conventional monomer/oligomer addition growth mechanism^30^, in this case by addition of silicic acid or silicic acid oligomers from solution, leading to the formation of increasingly silicified particles with increasing degree of condensation (Fig. 5a, b).

Increasing the polyamine concentration results in an increase in the number of positively charged amine molecules, which enhances the number of coacervates to particle nucleation (as the polyamine concentration takes longer to deplete). Hence adding polyamines results in a decrease in the ultimate particle size (Fig. 1b, c). To rationalize the influence of polyamine concentration on the size of the particles we applied equilibrium cluster theory developed by Groenewold and Kegel^56^, which relates particle size to the number of charges in solution. This model has been applied successfully to quantify cluster formation for several systems, including protein dispersions and colloid–polymer mixtures^54^.

For the equilibrium aggregation number n_*_ of an equilibrium cluster Groenewold and Kegel predict:^47^

1 $$n_ \ast = \frac{{2\gamma v}}{{Q\left( {z_0} \right)^2}}$$

with *γ* the surface tension between the particles and the solvent, *ν* the volume of primary aggregating particles, *Q* is the Bjerrum length and *z*_0_ is related to the particle surface charge density. In our experiments we only varied the polyamine concentration (*C*_polyamine_). Let us assume only *z*_0_ then varies and postulate *C*_polyamine_ ~ *z*_0_: adding more polyamine increases the number of surface charges per surface area. Since n_*_ is related to the final particle size *d* as n_*_ ~ *d*^3^, it follows:

2 $$d \propto \left( {C_{{{{{{{\mathrm{polyamine}}}}}}}}} \right)^{ - \frac{2}{3}}$$

We can also scale the different particle diameters with respect to each other to test the scaling result. Then we can relate particle diameter *d*_1_ at polyamine concentration *c*_1_ to particle diameter *d*_2_ at polyamine concentration *c*_2_ and write:^56^

3 $$\frac{{d_1}}{{d_2}} = \left( {\frac{{zc_1}}{{zc_2}}} \right)^{ - \frac{2}{3}}$$

**Fig. 6:**
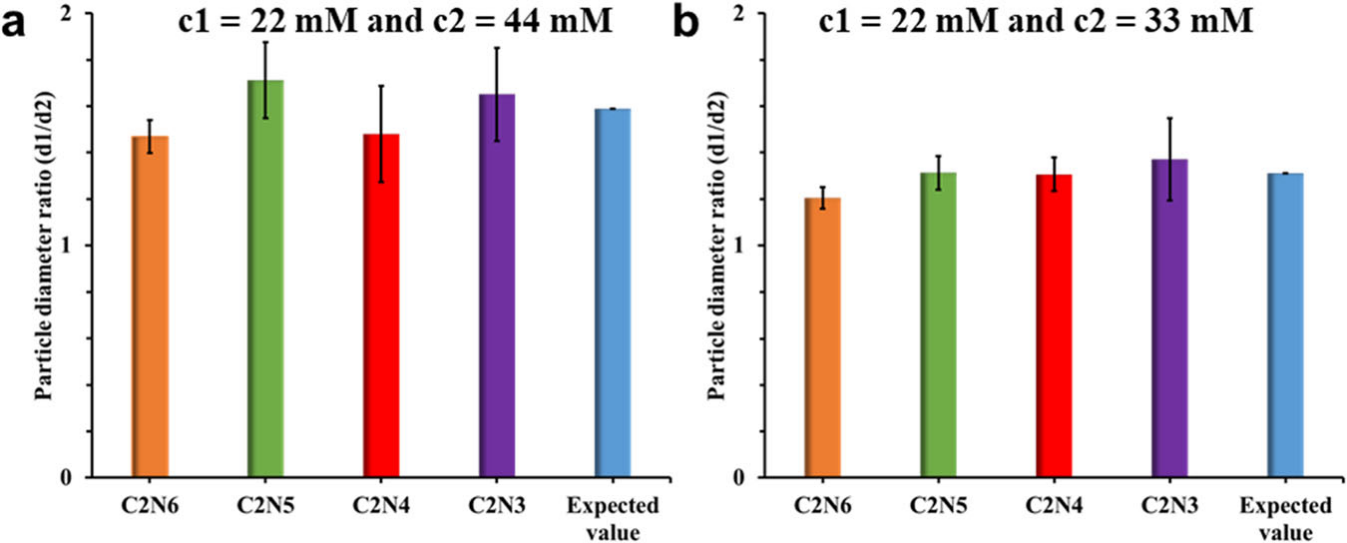
Ratio of particle diameter at different polyamine concentrations obtained using the Eq. (3). **a** Polyamine concentrations *c*_1_ = 22 mM and *c*_2_ = 44 mM. **b** Polyamine concentrations *c*_1_ = 22 mM and *c*_2_ = 33 mM (error bars represent standard deviation).

When the polyamine concentration is raised from 22 to 44 mM, the particle size is expected to decrease by 1.59 (Fig. 6), the observed decrease in particle size for all the amines was close to this value, demonstrating the polyamine concentration dependence of the size agrees well with the theoretical prediction (Fig. 6). Similar results were observed when the polyamine concentration was increased from 22 to 33 mM. The decrease in particle size with increasing polyamine concentration confirms the hypothesis that polyamine incorporation/depletion via coacervate formation is a key step in the nucleation of the particles.

While the role of polyamines on silicic acid condensation has been well established^14,15^, their effect on silica condensation is poorly understood. We therefore studied the effect of the incorporated polyamine on the overall degree of condensation of the silicic acid units (denoted *Q*^1^–*Q*^4^) in the co-precipitated particles with ^29^Si MAS NMR. Silica particles grown in presence of basic amino acids (under the similar reaction conditions and aqueous silicic acid concentration, resulting in particle diameter of 10–20 nm; Supplementary Note 2) do not incorporate the base molecules into their matrix^29,57^ and as a result, these particles typically exhibit high *Q*^4^/*Q*^3^ values^28,29,57^ in the range of 3.5–4.0 (75–80% *Q*^4^). In our particles grown in the presence of C_2_N_6_ at increasing TEOS to C_2_N_6_ mole ratios, a steady increase in *Q*^4^/*Q*^3^ values (Fig. 5a, b) was observed in ^29^Si NMR. This confirmed that the increased particle size that resulted from a higher TEOS addition also resulted in an overall increase in the final and irreversible condensation state Q^4^. A final *Q*^4^/*Q*^3^ ratio (Fig. 5b) of 1.5 (56% *Q*^4^) was obtained for the 617 ± 20 nm sized particles grown in presence of C_2_N_6_ (15T_1C_2_N_6_). Whereas *Q*^4^/*Q*^3^ ratio of 2.0 (Fig. 5b and Supplementary Fig. S10b) was obtained for the 137 ± 13 nm-sized particles produced in presence of C_2_N_3_ (15T_1C_2_N_3_). It can therefore be surmised that the presence of polyamines in general and C_2_N_6_ in particular; a strong decrease in the overall condensation of silica is observed. The strong correlation between the ammonium signal and the *Q*^3^ species (Fig. 5c and Supplementary Note 11) suggests an ionic interaction^15^ between the dissociated silanols and the protonated amine species. The strong correlation between *Q*^3^ silanols and amines is in agreement with the findings of recently published study^52^ on the interaction between polyamines and silica.

The results presented above demonstrate that silica particles grown in the presence of polyamines differ from simple base catalyzed silica particles. First, the polyamines have a profound effect on silica particle diameters, with particles exhibiting sizes larger than those grown in presence of simple organic and inorganic bases like l-lysine, ethylamine, and sodium hydroxide (Fig. 1 and Supplementary Fig. S2). Second, polyamines are incorporated into the silica particle matrix with greater polyamine localization in the particle core (Fig. 4b and Supplementary Fig. S7b), whereas bases like l-lysine are localized on the particle surface^28^. Third, the particles grown in the presence of polyamines are more poorly condensed with increasing polyamine length resulting in lower extent of condensation (Fig. 5a, b, and Supplementary Fig. 10c).

The above results point to a somewhat paradoxical property of polyamines: while aiding in the oligomerization of silicic acid^36^, they also prevent the final condensation of *Q*^3^ silica species to fully condensed *Q*^4^ species. While the particle synthesis conditions reported in this paper (60 °C and pH ≈ 11) differ considerably from those found in natural environments, the overall reduction in *Q*^4^/*Q*^3^ ratio of silica produced in the presence of polyamines lends *qualitative* support to the hypothesis that polyamines may also control silica condensation in biological environments^58^.

**Fig. 7:**
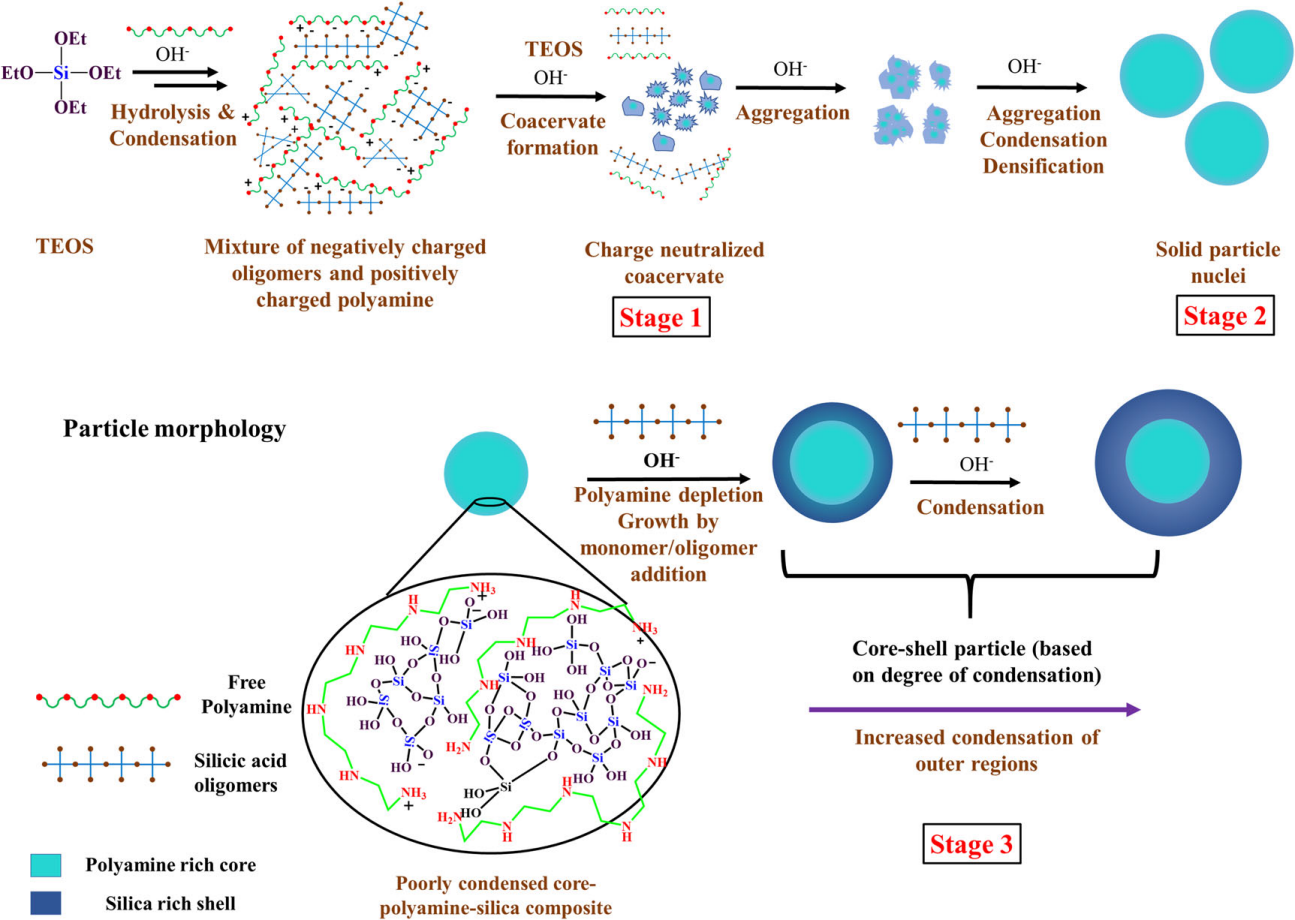
Schematic representation of silica particle growth in the presence of polyamines. The core–shell particle reflects the different degrees of condensation in the particle (green colour represents polyamine localization).

The overall particle nucleation and growth can now be depicted in three stages as shown in Fig. 7. (i) Oligomerization of silicic acid in solution, in intimate interaction with polyamines and with concomitant coacervate formation; further aggregation of these polyamine–silicic acid oligomer coacervates; (ii) Aggregation of coacervates, densification due to silanol condensation and above a critical aggregation size resulting in the formation of solid nuclei particles with accompanying densification by further condensation of the silicic acid oligomers into silica; (iii) Further “monomer/oligomer addition growth” of the solid particles with silicic acid oligomers after essential depletion of the polyamines from solution.

Silica particle synthesis in presence of polyamines results in (i) monodisperse particles with sizes ranging from 83 ± 15 nm (C_2_N_3_, Fig. 1b, c) to 617 ± 20 nm (C_2_N_6_, Fig. 1b, c); (ii) The particles contain embedded polyamines (Fig. 4); and (iii) silica condensation decreases with increasing polyamine length. Monodisperse silica particles reported in this study could find applications in the fields of drug delivery^59^, biosensing^60^, optical coatings^61^ and chromatography^62^. Polyamine-embedded silicas have been used as catalysts and in water remidiation^63^. Furthermore, by posttreatment polyamine-incorporated silicas can be further processed to improve hydrothermal stability of silica^64^. Recently^65^, it has been demonstrated that through the use of cyclams and methylated diamines silica particles can be assembled to produce tuneable hollow structures. Combining polyamine–silica particles with the above molecules might result in the production of novel functional materials.

## Conclusion

In summary, the effect of polyamine size on silica particle formation was studied using synthetic analogues of the polyamines found in diatoms. Our results prove that the polyamines have a very strong influence on the particle growth and the degree of silica condensation. Increasing polyamine length was found to result in an increase in the particle size, which can be attributed to the increased ability of longer polyamines to oligomerize and initiate early coacervate nucleation. Polyamines were incorporated into the silica particles, indicating that the final particles are co-localized polyamine–silica composites. In contrast to their known accelerating effect on silicic acid oligomerization (from *Q*^0^ to *Q*^3^), we demonstrate that the polyamines retard the final irreversible condensation of silica (conversion of *Q*^3^ to *Q*^4^) and that the longer polyamines are more efficient at reducing silica condensation than smaller polyamines. Based on the variation of silica condensation during particle growth and the development of the zeta potential, it is proposed that the final particles have a core–shell structure, with a polyamine-rich core and silica-rich shell.

## Methods

### Silica particle synthesis

Silica particle synthesis was carried out by modifying the method developed by Yokoi^29^ et al. The polyamine solutions were prepared fresh and were used within one day of preparation. The pH of freshly prepared aqueous polyamine solutions at 22 °C (room temperature) was 10.9. The particle synthesis was carried out using a biphasic reaction system, 15 mL of polyamine solution was added to 20 mL screw cap glass vials charged with 5 mm stirrer bar and heated to 60 °C on an aluminium block, while stirring at 900 rpm. The polyamine solution was equilibrated for 30 min at 60 °C to ensure that the temperature was stable. Thereafter, 1.1 mL of TEOS was added to reaction mixture and the stirring was continued for 16 h. After 16 h the particle dispersion was cooled to room temperature, centrifuged (12,000 rpm, 10 min), freeze-dried, and used for further analysis. The molar ratio of the reaction was 15 TEOS:*y*C_2_N_*x*_:2523 H_2_O where *y* = 1, 1.5 and 2; *x* = 3–6.

### Cryo-TEM

Vitrified thin films for CryoTEM analysis were prepared using an automated vitrification robot (FEI Vitrobot Mark IV) at 60 °C and 100% relative humidity by plunge vitrification in liquid ethane. Before vitrification the plunger pipet tips were cleaned using MilliQ water and 200-mesh copper grids covered with a Quantifoil R 2/2 holey carbon film (Quantifoil Micro Tools GmbH) were surface plasma treated for 40 s using a Cressington 208 carbon coater. CryoTEM imaging was carried out on the cryoTITAN (Thermo Fisher, previously FEI), equipped with a field emission gun (FEG), a post-column Gatan imaging filter (model 2002) and a post-GIF 2*k* × 2*k* Gatan CCD camera (model 794). The microscope was operated at 300 kV acceleration voltage in bright-field TEM mode with zero-loss energy filtering at nominal magnifications of ×6500 and ×24,000; both with a 1 s image acquisition time.

## Supplementary information

Supplementary information

## Data Availability

The data that support the findings of this study are available from the corresponding author upon reasonable request.
