# Supplementary material for: Chain length of bioinspired polyamines affects size and condensation of monodisperse silica particles
Source: Commun Chem. 2021 Nov 19;4:160. doi: 10.1038/s42004-021-00595-y (PMC9814531; doi:10.1038/s42004-021-00595-y)
Supplement: Supplementary file 1 — Supplementary information [file 42004_2021_595_MOESM1_ESM.pdf]

Supplementary Information

Chain length of bioinspired polyamines affects size and condensation of monodisperse silica particles

Sai Prakash Maddala [a,b], Wei-Chih Liao [c], Rick R. M. Joosten [a, b], Mohammad Soleimani [a, b] Remco Tuinier [a], Heiner Friedrich [a, b*] and Rolf A. T. M. van Benthem [a, c*]

[a]Laboratory of Physical Chemistry, Department of Chemical Engineering and Chemistry & Institute for Complex Molecular Systems, Eindhoven University of Technology, P.O. Box 513, 5600 MB Eindhoven, The Netherlands and [b]Center for Multiscale Electron Microscopy, Eindhoven University of Technology, Groene Loper 5, 5612 AE, Eindhoven, The Netherlands; [c]DSM Materials Science Center, 6167 RD Geleen, The Netherlands

*Corresponding authors:
Heiner Friedrich. Email: h.friedrich@tue.nl
Rolf A. T. M. van Benthem. Email: r.a.t.m.v.benthem@tue.nl

Supplementary Methods (Supplementary Note 1)

Materials: Tetraethyl orthosilicate (TEOS; ≥99%), Dietylenetriamine (C2N3; 99%), Triethylenetetramine (C2N4; ≥97%), Sodium hydroxide pellets (≥98%), Triethylamine (≥ 99.5%), L-lysine (≥ 98%) were purchased from Sigma Aldrich. Ethylamine (70% solution in water), Tetraethylenepentamine (C2N5) and Pentaethylenehexamine (C2N6) were purchased from TCI, Netherlands. All chemicals were used as received. Deionized water (Milli-Q®; Merck) was used for synthesis.

Silica particle synthesis with varying TEOS amounts: Silica particles were synthesized by modifying the method[1] developed by Yokoi *et al*. 22 mM polyamine solutions were heated to 60°C and equilibrated for 30 minutes. Different amounts of TEOS were added to the reaction mixture and the reactions were continued until the TEOS was completely hydrolysed. The reaction time[1,2] was chosen to ensure complete hydrolysis of TEOS and condensation of silicic acid to particles. The particle dispersions centrifuged

(12000 rpm, 10 minutes), and the isolated pellets were freeze-dried. The molar ratio of the reactions was z TEOS: 1C2Nx: 2523 H_2O where z = 0.90, 1.1, 3.4, 9.1, 11.4 and 15; x = 3, 5, and 6.

Characterization: The size of the silica particles was determined from SEM images of the particles. Briefly, 10 µL of the particle dispersion was deposited on a silicon wafer (preheated to 60°C) and dried at 60°C for 1 hour. The resultant film was then sputter-coated with platinum (Quorum Q150T plus turbo pumped sputter coater; UK) and imaged using SEM Quanta 3D FEG (TFS, USA), at an acceleration voltage of 20 kV. Average Silica particle diameter was determined using an in-house MATLAB script with 100 particles measured for each of the reaction conditions. For EDS elemental mapping, 50 µl of the silica particle suspension was dropped onto a standard 200 mesh copper TEM grid covered by a 10 nm continuous carbon film. TEM grids were placed in an in-house made sample holder suitable for an SEM stage. SEM-EDS mapping was performed using SEM Quanta 3D FEG (Thermo Fisher Scientific, USA), at an acceleration voltage of 10 kV. Zeta potential measurements were carried out on 1.5 mg/mL silica particle dispersions using Anton-Paar Litesizer 500 (Anton-Paar, Austria). TGA measurements (TA instruments, Germany) were carried out on freeze-dried particles. The 4 to 6 mg of freeze-dried silica particle powders were heated in platinum pans from 30°C to 1000 °C at 20°C/ min in air.

Solid-state NMR : Measurements were performed on an Oxford magnet of a static magnetic field at 9.4 T (operating frequency for ^{1}H and ^{29}Si at 400.1 MHz and 79.5 MHz, respectively) equipped with a Bruker NEO spectrometer. All samples were packed into either a 6 mm (o. d.) PENCIL rotor with two Teflon restrictors or a 1.6 mm (o. d.) PENCIL rotor and measured with Varian 6 mm or 1.6 mm HXY probe heads. Nitrogen gas was used to spin the rotor, and the temperature was regulated at 15°C during measurement to avoid further silica condensation. ^{29}Si and ^{1}H T_1 relaxation time constants were measured using saturation-recovery experiment. For 1D ^{29}Si MAS NMR spectra, magic-angle spinning (MAS) rate was set to 5 kHz, and the recycle delays, typically between 1200 and 2000 seconds, were set to 5 times of ^{29}Si T_1. High power proton decoupling was applied using SPINAL64 scheme[3] with a ^{1}H r.f. at ca. 62.5 kHz during signal acquisition. Some number between 110 and 150 of transients were averaged to afford spectra with a decent signal-to-noise ratio. For spectra processing 50 Hz of apodization was applied. The ^{29}Si chemical shift is externally referenced to tetramethylsilane (TMS) at 0 ppm. SSNake software[4] was used to afford spectra fitting result. For 1D ^{1}H MAS NMR spectrum, MAS rate was set to 30 kHz, and the recycle delay was set to 5 times of ^{1}H T_1. For 2D ^{29}Si-^{1}H heteronuclear correlation (HETCOR) experiments, a ramped cross polariton (CP) scheme was used with a ramp profile between 90% and 100 % on ^{1}H. MAS rate was set to 30 kHz, and the recycle delay was set to 1.3 times of ^{1}H T_1. CP contact times were set to 0.1 ms or 3 ms , and

high power proton decoupling was applied using SPINAL64 scheme[3] with ^{1}H r.f. power at ca. 100 kHz during signal acquisition. 8192 and 512 transients were averaged per *t1* increment for short and long CP contact times, respectively. STATES-TPPI method was used, and 16 *t1* increments were collected. 200 Hz and 10 Hz of apodization were applied on ^{29}Si and ^{1}H dimension, respectively.

Supplementary Note 2: Cryo-TEM images of particles produced at pH 11 using simple bases

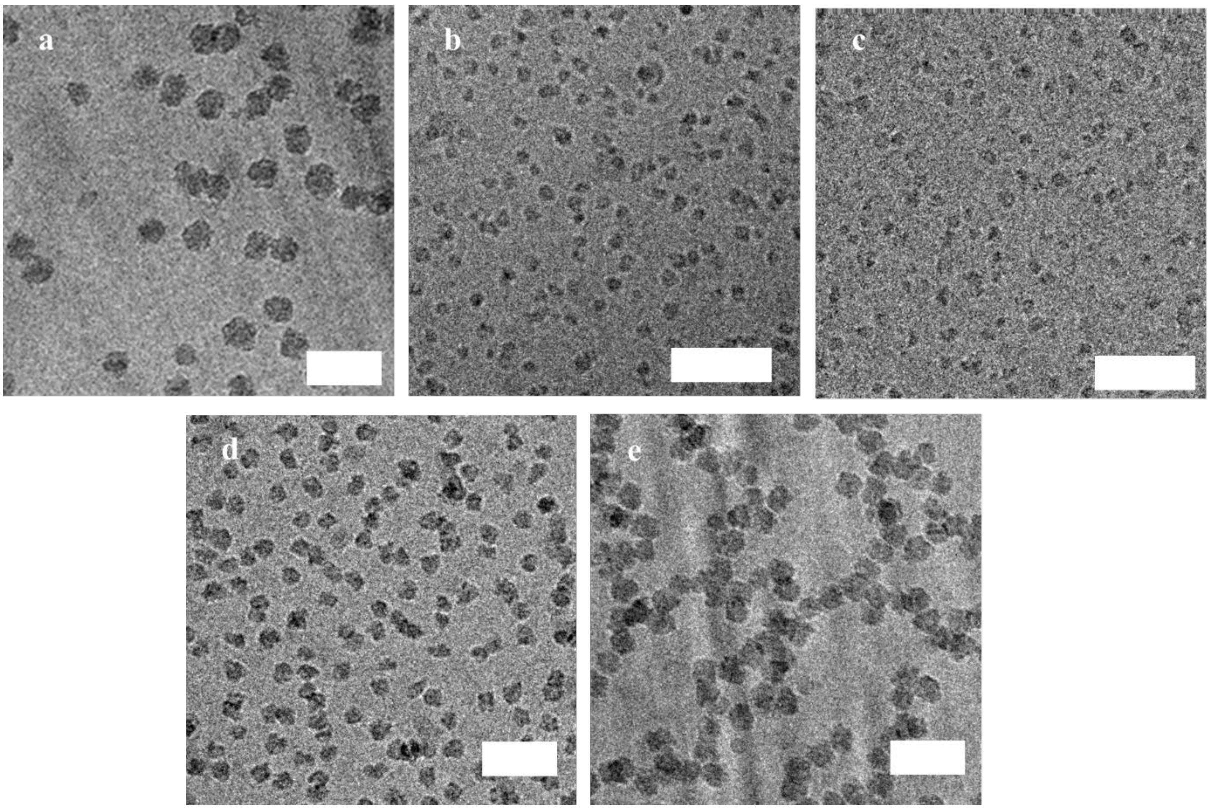

Figure S2: Cryo-TEM images of silica particles produced in presence of simple bases at initial pH of 11; initial pH measured at room temperature. (a) NaOH; (b) Ethylamine (44 mM); (c) Triethylamine (44mM); (d) L-Lysine (22mM); (e) L-Lysine (44 mM) [Scale bar 50 nm].

Supplementary Note 3: Additional SEM images of silica particles synthesized at different polyamine concentrations

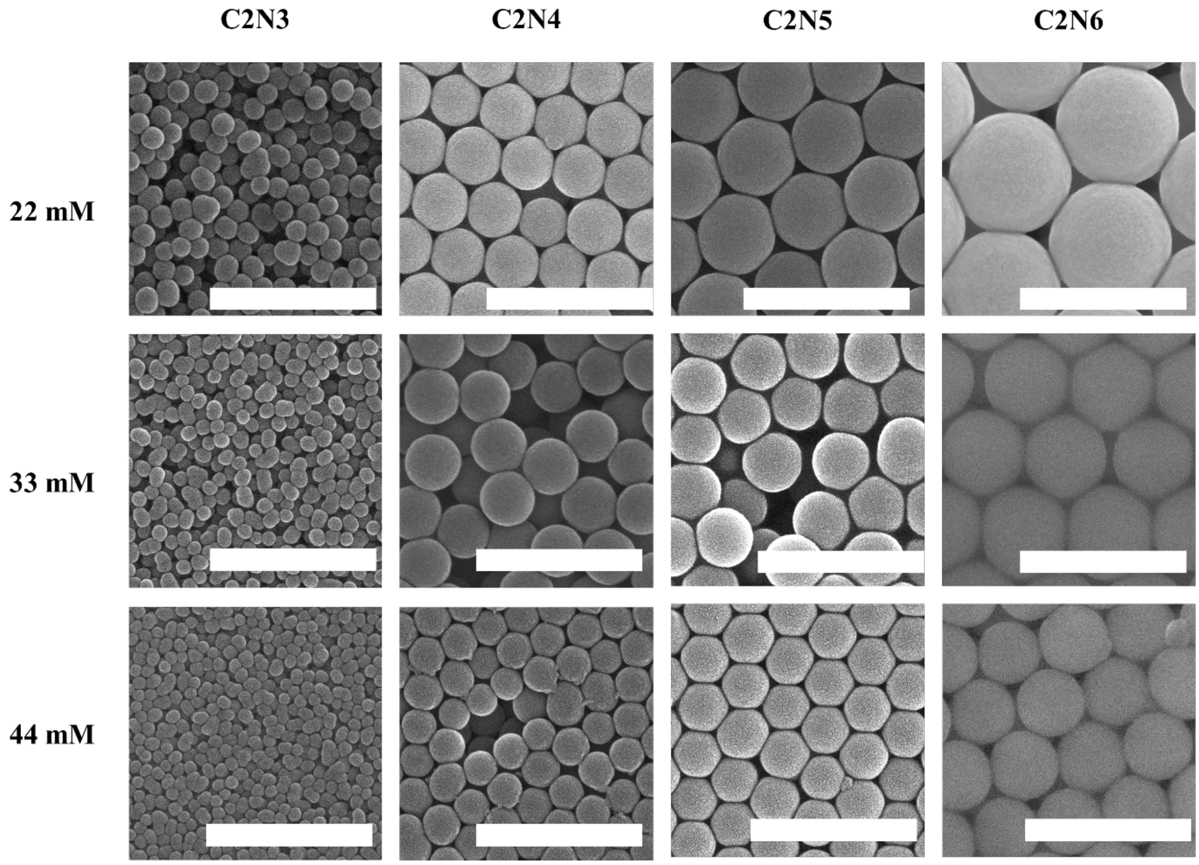

Figure S3: SEM images of silica particles synthesized at different polyamine concentrations (Scale bars: 1 µm). The reactions were carried out overnight.

Table S3: Size of silica particles produced in presence of different polyamines. Average Particle diameters (± Standard deviation) were measured using in-house MATLAB script, 100 particles were measured for each sample.

Polyamine	22 mM (Polyamine conc.)	33 mM (Polyamine conc.)	44 mM (Polyamine conc.)
**Diethylenetriamine (C2N3)**	137±13 nm	100±15 nm	83±15 nm
**Triethylenetetramine (C2N4)**	333±17 nm	255±13 nm	225±17 nm
**Tetraethylenepentamine (C2N5)**	479±16 nm	365±23 nm	280±17 nm
**Pentaethylenehexamine (C2N6)**	617±20 nm	512±17 nm	420±27 nm

Supplementary Note 4: Effect of TEOS concentration on silica particle size at constant polyamine concentration

Table S4a: Average particle diameters of silica particles produced at varying TEOS_Polyamine mole ratios. Average Particle diameters (± standard deviation) were measured using in-house MATLAB script, 100 particles were measured for each sample.

Sample id	C2N3	C2N5	C2N6
**0.9T_1C2N*x***	70±7 nm	235±35 nm	368±56 nm
**1.1T_1C2N*x***	68±6 nm	248±25 nm	358±41 nm
**3.4T_1C2N*x***	74±8 nm	316±17 nm	381±43 nm
**9.1T_1C2N*x***	102±11 nm	418±29 nm	511±35 nm
**11.4T_1C2N*x***	108±12 nm	429±46 nm	516±37 nm
**15T_1C2N*x* (Final concentration)**	137±13 nm	479±16 nm	617±20 nm

Predicted particle diameter: Predicted particle diameter was determined using the following equation (Supp. Eq. 1):

$$Predicted\ diameter = Final\ diameter \times \frac{Silicic\ acid\ concentraton}{Final\ Silicic\ acid\ concentration}$$

Final Silicic acid concentration = 330 mM

Silicic acid concentration at various zTEOS_1C2Nx mole ratios is given below in Table S4b

Table S4b: Silicic acid concentrations at various mole ratios.

Sample id	Silicic acid concentration
**0.9T_1C2N*x***	20 mM
**1.1T_1C2N*x***	25 mM
**3.4T_1C2N*x***	75 mM
**9.1T_1C2N*x***	200 mM
**11.4T_1C2N*x***	250 mM
**15T_1C2N*x* (Final concentration)**	330 mM

Supplementary Note 5: SEM images of silica particles produced at low concentration

The SEM samples were produced by depositing 10 µL sample on silicon wafer preheated to 60°C. These sample preparation steps were adopted to avoid artefacts that might result from cooling the reaction.

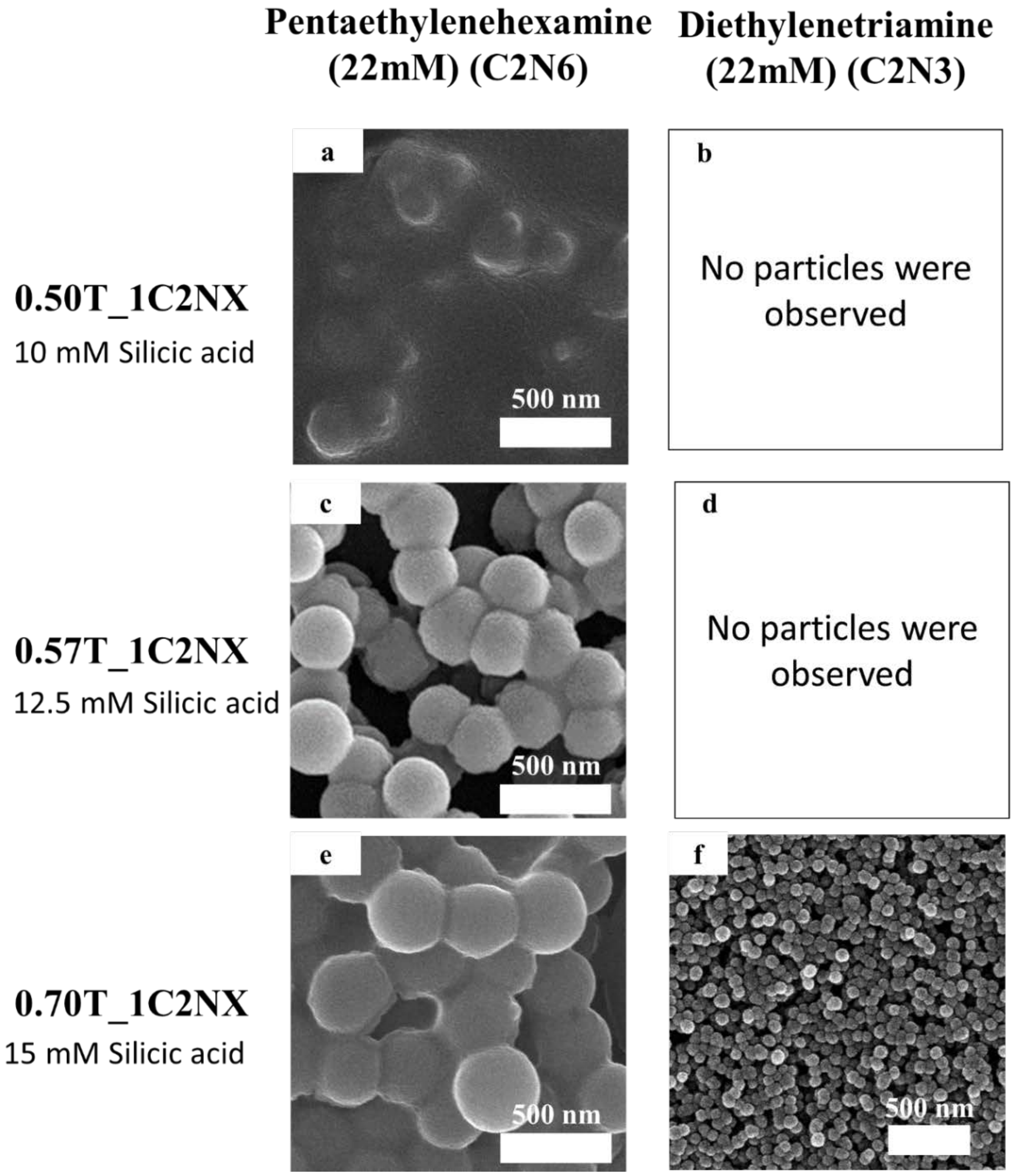

Figure S5: a) c) and e) SEM images of silica oligomer aggregates at 0.50TEOS_ 1C2N6 mole ratio, and particles at 0.57 and 0.70TEOS_1C2N6 mole ratios respectively; **b)** and **d)** particles were not observed at 0.50 and 0.57TEOS_1C2N3 mole ratio; **f)** SEM images of particles observed at 0.70TEOS_ 1C2N3.

Supplementary Note 6: Cryo-TEM silica particles produced at presence of C2N3(Diethylene triamine) at 0.70TEOS_1C2N3– Time course measurement

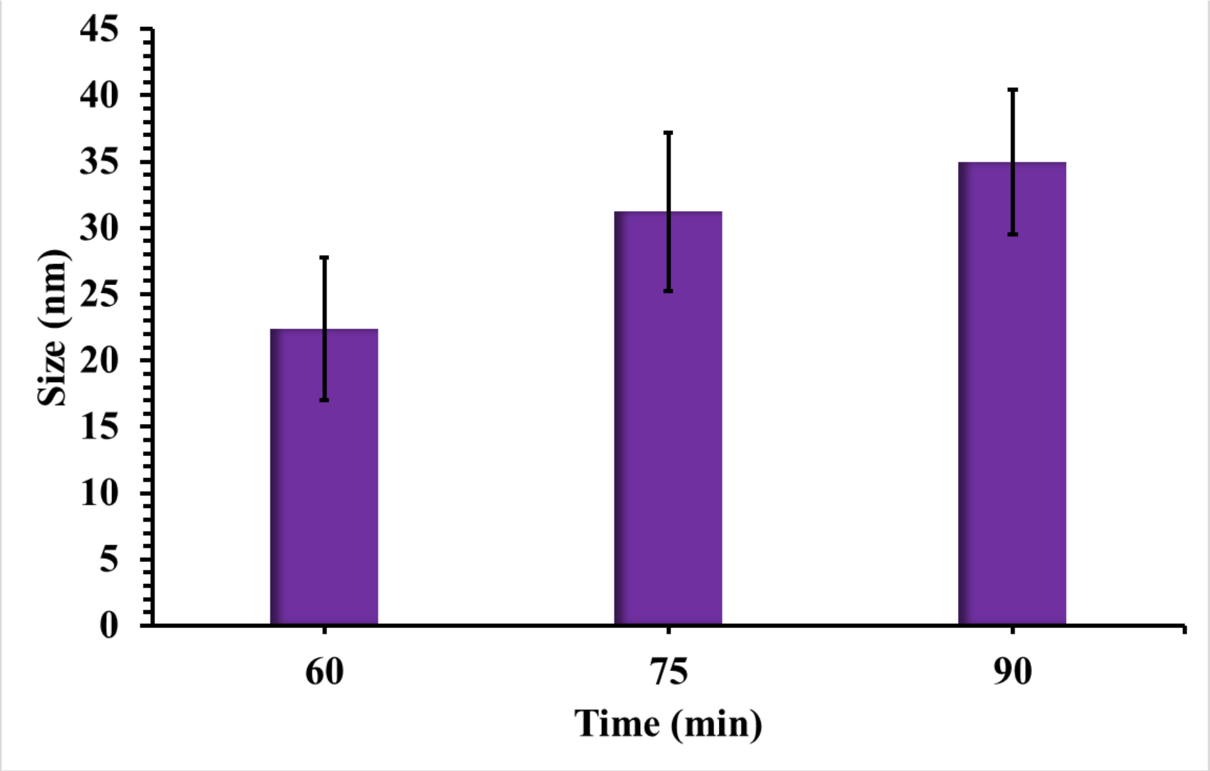

Figure S6: Average particle diameter of silica particles produced in presence of 22 mM C2N3 at 0.70TEOS_1C2N3 mole ratio; Average particle diameter determined from Cryo-TEM images by measuring 100 particles (error bars represent standard deviation).

Supplementary Note 7: Energy Dispersive X-Ray spectroscopy (EDS)

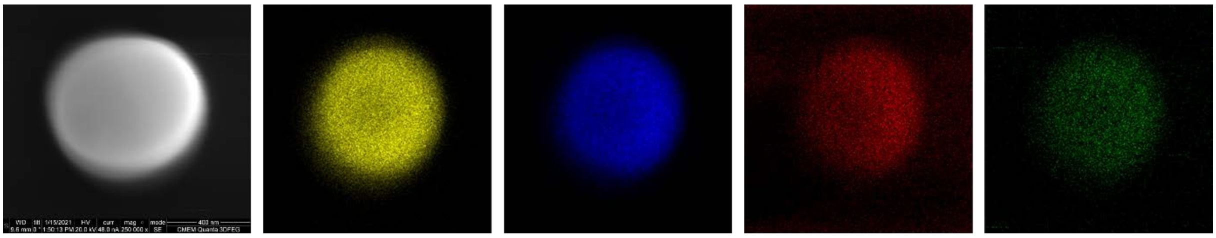

SEM image | Silicon | Oxygen | Carbon | Nitrogen

Figure S7a: SEM-EDS map of silica particle produced in presence of C2N6 at 15T_1C2N6 mole ratio showing the presence of carbon and nitrogen along with silicon and oxygen indicating co-localization of polyamines with silica.

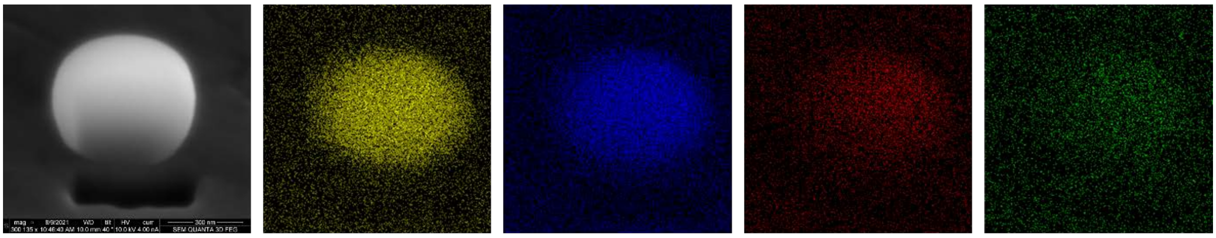

FIB-SEM micrograph | Silicon | Oxygen | Carbon | Nitrogen

Figure S7b: SEM-EDS map of FIB milled silica particle produced in presence of C2N6 at 15T_1C2N6 mole ratio showing the presence of carbon and nitrogen along with silicon and oxygen indicating internal co-localization of polyamines with silica.

Supplementary Note 8: Infra-Red spectroscopy

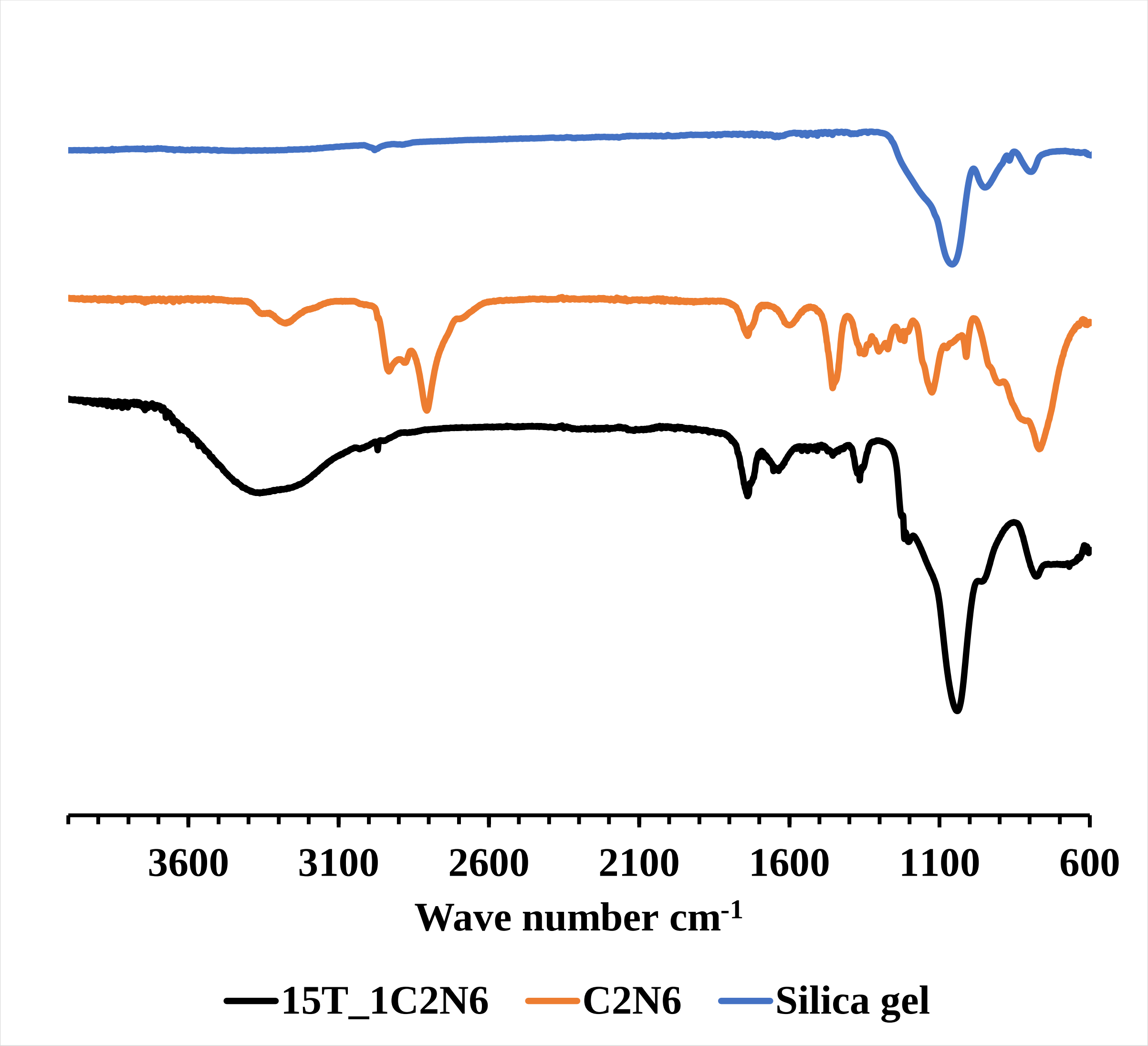

Figure S8: FTIR spectrum of C2N6 (Orange) and freeze dried 617 ± 20 nm silica particles grown in presence of 22 mm C2N6 (black; 15T_1C2N6). FTIR spectrum of silica produced in absence of polyamines (blue) provided for reference.

Supplementary Note 9: Thermo Gravimetric Analysis

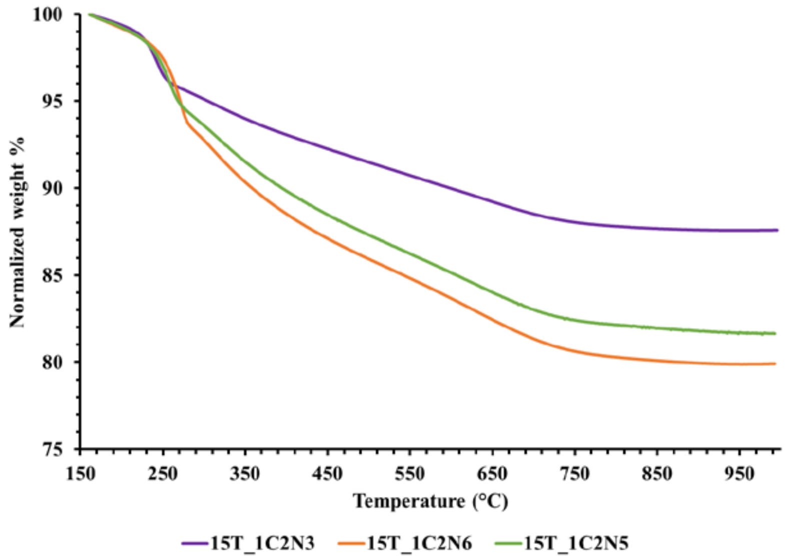

Figure S9: Normalized TGA curves of silica particles grown at 15TEOS_1C2N*x* mole ratio obtained by heating the samples in air. Samples were heated from RT to 1000°C at 20°C/min. Sample weight-loss was normalized from 160°C to account for adsorbed water.

Supplementary Note 10: 1D NMR Spectra

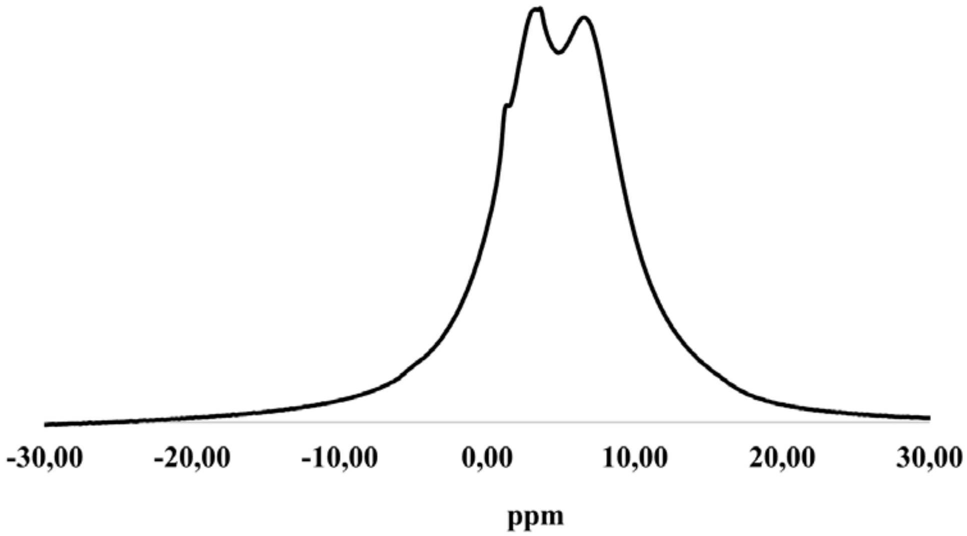

Figure S10a: ^{1}H NMR spectrum of silica particles produced at 15T_1C2N6 mole ratio.

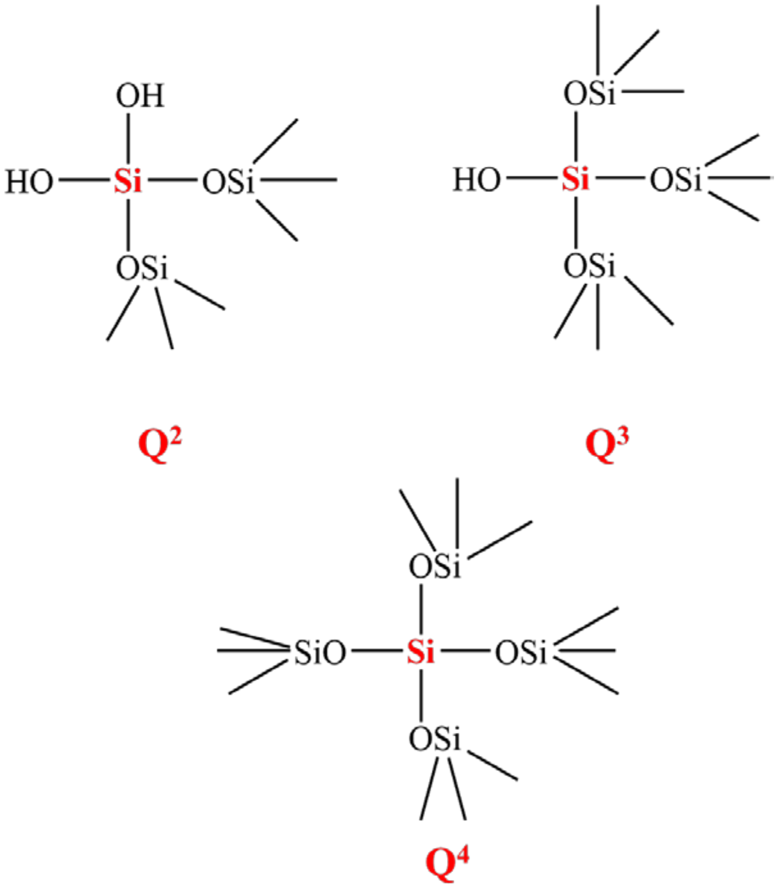

Figure S10b: Silicon environments (in red) identified from ^{29}Si MAS spectra of freeze-dried silica particles.

Table S10: ^{29}Si Chemical shifts and relative amounts obtained from quantitative ^{29}Si MAS NMR

Sample	Q4 ppm (%)	Q3 ppm (%)	Q2 ppm (%)
0.9T_1C2N6	-110 ppm (47.3%)	-100 ppm (41.4%)	-92.5 ppm (11.3%)
3.4T_1C2N6	-110 ppm (49.5%)	-100 ppm (42.4%)	-90.6 ppm (8.1%)
9.1T_1C2N6	-110 ppm (52.7%)	-100 ppm (40.6%)	-90.6 ppm (6.8%)
15T_1C2N6	-110 ppm (55.9%)	-100 ppm (38.1%)	-91.2 ppm (6.0%)
15T_1C2N3	-111 ppm (63.2%)	-100 ppm (32.4%)	-90.9 ppm (4.4%)

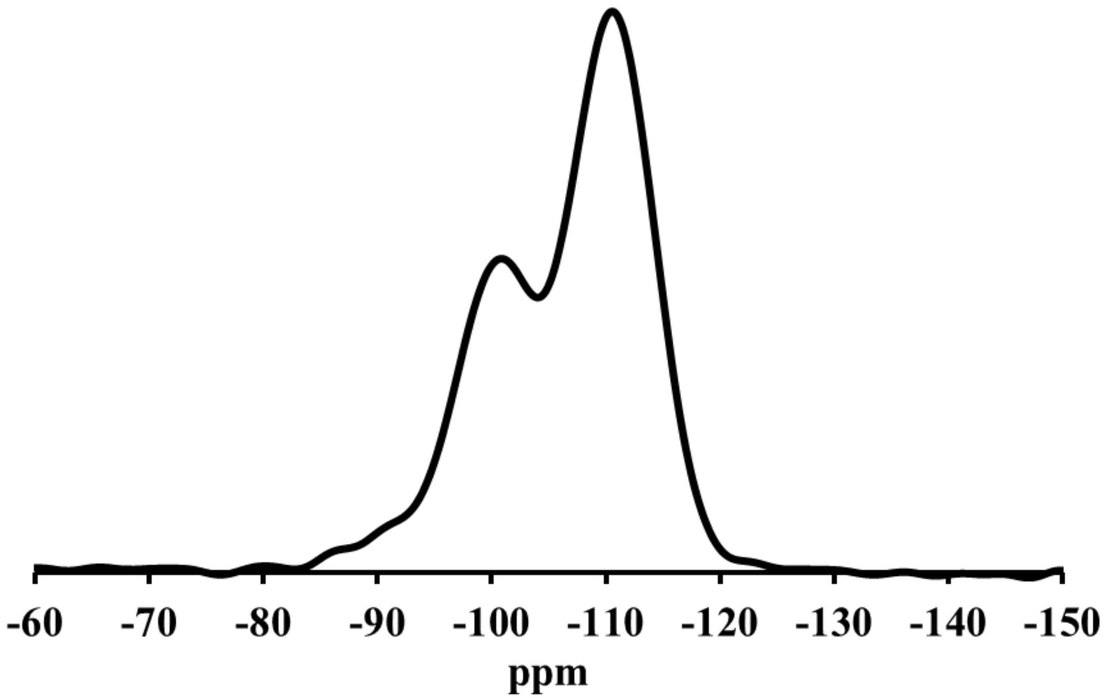

Figure S10c: ^{29}Si NMR of silica particles produced at 15T_1C2N3 mole ratio.

Supplementary Note 11: 2D ^{29}Si-^{1}H HETCOR

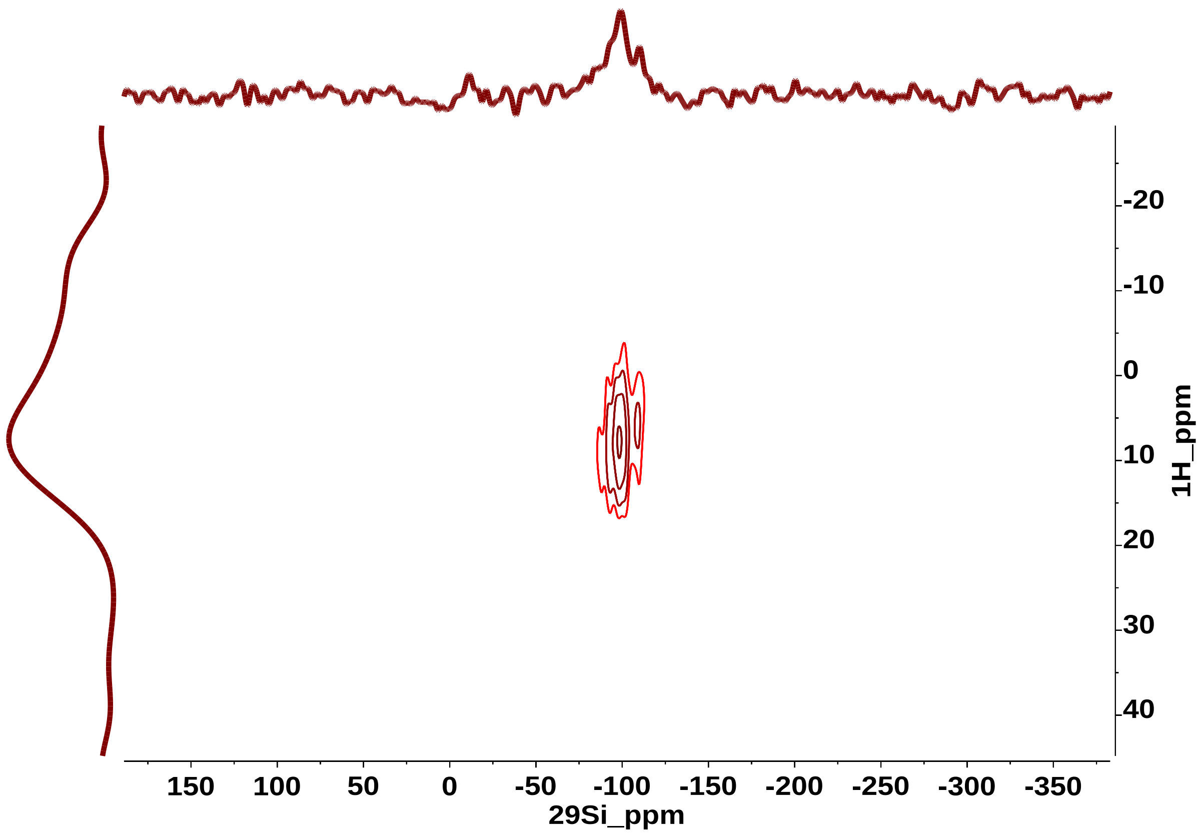

Figure S11: 2D ^{29}Si-^{1}H HETCOR NMR with CP contact time of 0.1 ms of silica particles produced at 15T_1C2N6 mole ratio.

Supplementary Note 12: pKa values of amines

Table S12: pKa values of the polyamines.

Polyamine	pKa 1	pKa 2	pKa 3	pKa 4	pKa 5	Ref
Ethylamine	10.8					5
Triethylamine	10.8					5
Ethylene diamine	10.1	7.0				5
Diethylene triamine	9.9	9.1	4.3			6
Triethylene tetramine	9.9	9.2	6.7	3.3		6
Tetraethylene pentamine	9.9	9.1	7.9	4.3	2.7	6

References

1 Yokoi, T. *et al.* Mechanism of Formation of Uniform-Sized Silica Nanospheres Catalyzed by Basic Amino Acids. *Chemistry of Materials* **21**, 3719-3729, doi:10.1021/cm900993b (2009).

2 Fouilloux, S., Tache, O., Spalla, O. & Thill, A. Nucleation of silica nanoparticles measured in situ during controlled supersaturation increase. Restructuring toward a monodisperse nonspherical shape. *Langmuir* **27**, 12304-12311, doi:10.1021/la2013842 (2011).

3 Fung, B. M., Khitrin, A. K. & Ermolaev, K. An improved broadband decoupling sequence for liquid crystals and solids. *J Magn Reson* **142**, 97-101, doi:10.1006/jmre.1999.1896 (2000).

4 van Meerten, S. G. J., Franssen, W. M. J. & Kentgens, A. P. M. ssNake: A cross-platform open-source NMR data processing and fitting application. *J Magn Reson* **301**, 56-66, doi:10.1016/j.jmr.2019.02.006 (2019).

5 Ian S. Blagbrough, Abdelkhader A. Metwally., and Andrew J. Geall. in *Polyamines Methods and Protocols* (ed Jr. Anthony E. Pegg Robert A. Casero) 493-503 (Humana Press, 2011).

6 Vesterberg, O. Synthesis and Isoelectric Fractionation of Carrier Ampholytes. *Acta Chemica Scandinavica* **23**, 2653-2666, doi:DOI 10.3891/acta.chem.scand.23-2653 (1969).
